# Preparation, structural characterization, antioxidant activity and protection against cisplatin-induced acute kidney injury by polysaccharides from the lateral root of Aconitum carmichaelii

**DOI:** 10.3389/fphar.2022.1002774

**Published:** 2022-10-20

**Authors:** Maoying Tian, Lin Wang, Zhaowei Dong, Xi Wang, Xiaoyan Qin, Chao Wang, Jin Wang, Qinwan Huang

**Affiliations:** ^1^ State Key Laboratory of Southwestern Chinese Medicine Resources, College of Pharmacy, Chengdu University of Traditional Chinese Medicine, Chengdu, China; ^2^ Sichuan Integrated Traditional Chinese and Western Medicine Hospital, Chengdu, China

**Keywords:** polysaccharides, response surface methodology, oxidative stress, anti-apoptosis, ferroptosis, Aconitum carmichaelii

## Abstract

Response surface methodology (RSM) and Box- Behnken design (BBD) based on one-way experiments were used to optimize the extraction parameters of the lateral root polysaccharides of Aconitum carmichaelii. The extracted polysaccharides were named as refined fucose polysaccharide. The optimal conditions included a water to raw material ratio of 43, an extraction time of 2 h, and an extraction temperature of 90°C. The shape of RFP was shown by infrared spectroscopy (IR) and scanning electron microscopy (SEM) analysis. The monosaccharide composition and molecular weight of RFP was determined by high-performance liquid chromatography (HPLC). Furthermore, RFP exhibited moderate antioxidant activity by analyzing the scavenging rates of 2,2-diphenyl-1-picrylhydrazyl radical, superoxide anion radical, hydroxyl radical, and ABTS + radical. RFP exerted cytoprotective effects against hydrogen peroxide (H_2_O_2_)-induced injury in the rat renal tubular epithelial cell line rat renal tubular epithelial cells (NRK-52E) and inhibited apoptosis. In addition, researches found that RFP could alleviate cisplatin-induced acute kidney injury in mice by enhancing the levels of glutathione (GSH) and glutathione peroxidase-4 (GPX-4), decreasing the levels of malondialdehyde (MDA) and 4-hydroxynonenal (4-HNE), reducing lipid peroxidation, and thus inhibiting ferroptosis. In conclusion, this study provides a good strategy for obtaining bioactive polysaccharides from Fuzi.

## 1 Introduction

*Aconitum carmichaelii* is a famous traditional Chinese medicinal herb that is mainly distributed in Sichuan, Shaanxi, Hubei, Hunan, Yunnan provinces of China. The dried lateral root of *Aconitum carmichaelii* called “Fuzi” or monkshood in Chinese or “Bushi” in Japan and “Kyeong-Po Buja” in Korea, has been used as a traditional Chinese Medicine for more than 1,000 years for treating shock resulting from low blood pressure, acute myocardial infarction, coronary heart disease, chronic heart failure (Zhou et al., 2015; Fu et al., 2022a). In addition, it is also used in medicinal dishes, medicinal wines and teas in the Guangdong region of China or in Southeast Asia. To date, the more studied chemical constituents of epiphyllum are the alkaloid constituents. The C19 and C20 alkaloids are considered to be the main active and toxic constituents in epiphyllum. In addition to alkaloids, it also contains flavonoids, polysaccharides, sterols, organic acids and other chemical components (Fu et al., 2022b). Recent research have proved Fuzi possesses multiple pharmacological effects of protecting the cardiovascular system (Zhao et al., 2012), immune system (Konno et al., 1985), anti-inflammation and analgesia, anti-tumor (Zhang et al., 2017), anti-aging (Wang et al., 2012; Tong et al., 2013), hypoglycemia, hypolipidemia and protecting kidney and regulating energy metabolism. In traditional Chinese medicine theory, it is believed that Fuzi is mainly attributed to the kidney meridian and has the effect of tonifying fire and helping Yang, which is used in the evidence of dead Yang crisis. Modern studies have found that its aqueous decoction has the pharmacological effect of improving kidney damage in rats with microscopic lesions of kidney disease and protecting the kidneys in mice with a chronic renal failure model of kidney yang deficiency (Yang et al., 2010). In clinical practice, Fuzi and its group formulas are often used to promote the treatment of chronic kidney disease. Zhu Liangchun, a master of national medicine, treated chronic nephritis in which there was a deficiency of Yang in the spleen and kidneys, flooding of water and dampness, or even severe oedema of the face and limbs, by adding and subtracting it and Ji sheng Ren qi Wan, and by treating heavily with it in doses up to 30–60 g (Zhu and Cheng, 2019). Zhen Wu Tang is a representative formula for the treatment of yin and water caused by the deficiency of kidney yang of Fuzi, and is mainly used to treat chronic diseases such as chronic renal failure, chronic nephritis, nephrotic syndrome, lupus nephritis and diabetic nephropathy (Hao, 2018). In addition, the extracted polysaccharide from the concoction of it can effectively improve the status of mice with chronic kidney disease through the hypothalamic-adrenal axis (Wang et al., 2019).

Before the 20th century, it was believed that the active ingredient of Fuzi was alkaloids. Until 1986, Konno reported that four polysaccharides, which had hypoglycemic effects, were isolated in Japan (Chi et al., 2006). Combined with the various clinical applications of Fuzi and some pharmacological effects that cannot be wholly explained by alkaloids, another type of biologically active ingredient-polysaccharide was likely to play an important role. Consequently, researches on polysaccharides from Fuzi were studied. It was shown to have effects of anti-depression (Yan et al., 2010), protecting cardiomyocytes (Liao et al., 2013), regulating glucose and lipid metabolism (Huang et al., 2010), antibacterial (Lin et al., 2011), immune stimulation, and anti-tumor with few side effects (Chi et al., 2006).

Cisplatin is a widely used drug for oncology treatment, however, approximately 25% of patients using cisplatin sustain renal injury (Ikeda et al., 2021). Cisplatin is now found to cause acute kidney injury through several mechanisms, such as increased oxidative stress (Guo et al., 2018), promoting inflammatory responses (Gwon et al., 2021), and causing renal vascular injury (Martin-Sanchez et al., 2017). More recent experiments have shown that ferroptosis is involved in several models of acute kidney injury and that the accumulation of intracellular iron content and lipid peroxide affects the occurrence of ferroptosis, so attenuating the occurrence of ferroptosis in kidney cells could provide a new avenue for the treatment of chronic kidney disease. Many natural plant-derived components such as curcumin reduced cisplatin-mediated inflammation and oxidative stress by inhibiting the Toll like receptor 4 (TLR4)/Nuclear factor kappa B (NF-κB) axis and activating the cytoprotective enzyme heme oxygenase 1 (Guerrero-Hue et al., 2019),6-gingerol inhibited the expression of the receptor interacting protein kinase-1 (RIPK1), the receptor interacting protein kinase-3 (RIPK3), and phosphorylation of mixed lineage kinase domain-like protein (MLKL) in cisplatin-treated renal tissues, which in turn inhibited renal tubular cell necrosis (Gwon et al., 2021). Polydatin, a thujaplicin, affected cellular ferroptosis by stabilizing the Xc^−^ -GSH-GPx4 axis and iron metabolism (Zhou et al., 2022), and pine bark extract ameliorated cisplatin-induced acute kidney injury by increasing antioxidant enzyme activity and inhibiting lipid peroxidation (Lee et al., 2017). All of the above compounds were effective in ameliorating cisplatin-mediated renal injury or affecting cellular ferroptosis. However, to our best knowledge, there is no study on the amelioration of cisplatin-induced acute kidney injury by refined fucose polysaccharide (RFP).

In this study, an extraction method of RFP was screened and its structural features were elucidated: including molecular weight, monosaccharide composition, molecular structure, and glycosyl linkage. In addition, the *in vitro* activity of RFP was elucidated, including the capacity of RFP to scavenge 1,1-diphenyl-2-picrylhydrazyl (DPPH), hydroxyl, superoxide anion, and 2,2′-azino-bis-(3-ethylbenzothiazoline-6-sulfonic acid) ABTS + radicals, its antimicrobial capacity, as well as its effect on NRK-52E cells. Finally, the protective effect of RFP on cisplatin-mediated acute kidney injury in mice was explored on this basis. The above results provide a basis for further development of RFP utilization.

## 2 Materials and methods

### 2.1 Reagents

Fuzi was collected from the standardized cultivation demonstration base of Fuzi in Jiangyou County, Sichuan, China, and identified by Prof. Huang Qinwan (Chengdu University of Traditional Chinese Medicine). The voucher specimen (No. 100190501) is stored in the herbarium of the Chinese Medicine Processing Laboratory of Chengdu University of Chinese Medicine. Then, fuzi was dried at 50°C, powered and stored at room temperature. DPPH, hydrogen peroxide (H_2_O_2_), EDTA-2Na, ascorbic acid, salicylic acid, FeSO4, pyrogallol and ABTS+ were purchased from Sigma Chemicals Co. Rhamnose, D-galacturonic acid, D-glucose, D-galactose, xylose, and L-arabinose were purchased from Chengdu Pfeiffer Biotechnology Co (Chengdu, China). Cell proliferation assay kit (MTT). MTT cell proliferation assay kits were purchased from Multi Sciences Biotech, Co. (Hangzhou, Zhejiang, China), and high-sugar Dulbecco’s modified Eagle medium (DMEM) and fetal bovine serum (FBS) were purchased from Gibco Laboratories (Grand Island, NE, United States). Trypsin, penicillin, and streptomycin were purchased from Invitrogen (Carlsbad, CA, United States). Cisplatin was purchased from Hefei Chisheng Biotechnology Co., Ltd. (Hefei, China). Ferrostain-1(Fer-1) was purchased from GLPBIO (Shanghai, China). Creatinine (Cr), urea nitrogen (BUN), total protein (Komas Brilliant Blue method), malondialdehyde (MDA), tissue iron, reduced glutathione (GSH), superoxide dismutase (SOD) assay kit and mouse GPX-4 ELISA kit were purchased from Nanjing Jiancheng Institute of Biological Engineering (Nanjing, China), and bicinchoninic acid (BCA) protein concentration assay kit (enhanced) was purchased from Biyuntian Ltd. (Shanghai, China), and mouse 4-HNE ELISA kit was purchased from Elite Biotechnology (Wuhan, China). All other chemicals and reagents used in the experiments were of analytical grade.

### 2.2 Extraction of crude polysaccharides

The polysaccharide components of natural plants are polar macromolecules that dissolve easily in water, so hot water was used to extract polysaccharides in this study (Huang and Huang, 2020). A simple one-factor examination experiment was first carried out. Fuzi powder was extracted at different extraction times (1, 1.5, 2, 2.5, 3 h), extraction temperatures (60, 70, 80, 90, 100°C) and ratios of water to raw material (20:1, 30:1, 40:1, 50:1, 60:1). The above procedures were repeated twice. Then, combined filtrates were centrifuged at 3,500 rpm for 15 min. The supernatant was concentrated to 0.1 g/ml using a rotary evaporator. Then the supernatant was precipitated overnight at 4°C with four volumes of 95% ethanol. The crude polysaccharides precipitate was dried using a vacuum freeze-dryer. The contents of the polysaccharides were measured by the phenol-sulfuric method as previously reported (Du et al., 2016). Glucose was used as the standard and the yield (%) of polysaccharides (RFP) was calculated using Equation b:

$$Yield\;\left(\%\right)=m_{1}/m_{0}$$ (b)

where m_1_ was the content of crude polysaccharides (g), and m_0_ was the weight of dried Fuzi.

### 2.3 Response surface methodology design

Based on the preliminary single-factor experiments, a three-factor Box–Behnken design was employed to obtain the experiment design, statistical analysis results, and regression model. The entire design contains 17 random-sequence experimental runs. The three levels of each variable (low, medium, and high values) are encoded as -1, 0, and 1, respectively. In order to optimize the extraction conditions, a second-order polynomial formula is used to represent the response (polysaccharide yield) as a function of the variable, which is shown below (c):

$$Y=k_{0}+k_{1}A+k_{2}B+k_{3}C+k_{4}AB+k_{5}AC+k_{6}BC+k_{7}A^{2}+k_{8}B^{2}+k_{9}C^{2}$$ (c)

where Y is the predicted response, k_0_ is the constant coefficient, k_1_, k_2_, and k_3_ are linear coefficients, k_4_, k_5_, and k_6_ are interaction coefficients and k_7_, k_8_, and k_9_ are quadratic coefficients, A, B, C are independent variables.

### 2.4 Extraction of refined polysaccharides from RFP

The crude polysaccharide also contains other ingredients such as starch, protein, pigments, and small molecules, which should be removed. First, crude polysaccharides powder was dissolved in distilled water containing 1% (m/v) α-amylase at 60°C in a water bath for 3 h. Thereafter, 0.1% (m/v) papain was added to the solution at 40°C and pH 6 for 2 h. Protein was removed using the Savege method (chloroform-butanol = 4:1 v/v). The polysaccharide solution was decolorized with activated carbon, and subjected to dialysis in a dialysis bag (molecular interception = 3,500 KD) in distilled water for 3 days. After the dialysis, the solution was freeze-dried, then, RFP was obtained.

### 2.5 Chemical analysis of crude ABPS

The extracted polysaccharides were analysed for total sugars using the phenol-sulphuric acid method using anhydrous glucose as a standard (DuBois et al., 1956). Protein content was determined by a BCA kit using bovine serum albumin (BSA) as a standard. The uronic acid content was quantified by the method described by Blumenkrantz and Asboe-Hansen (1973) using galacturonic acid as a standard.

### 2.6 Molecular weight

The molecular weight of RFP was measured by high-performance gel permeation chromatography using a Shimadzu HPLC system equipping with GPC columns (BoRui Saccharide BRT105-104-102 8 × 300 mm). The calibration curve was plotted by a series of dextran with molecular weights of 5, 11.6, 23.8, 48.6, 80.9,148, 273, 409.8, and 667.8 kDa, respectively. The mobile phase was 0.05 mol/L NaCl, the column temperature was 40°C, the flow rate was 0.6 ml/min, and the injection volume was 20 μl.

### 2.7 Monosaccharides composition analysis

Monosaccharide composition of RFP was determined by HPLC as previously described (Wang et al., 2019). FRP (20 mg) was hydrolyzed by 2 ml of sulfuric acid (2 M) at 110°C for 6 h. Then the solution was neutralized with NaOH (8 M), and then diluted to 5 ml with water. Subsquently, 200 μl of hydrolysate was added to 200 μl PMP solution (0.5 M in methanol) and 200 μl of NaOH (0.3 M). The mixture was incubated at 70°C for 30 min, and neutralized with 200 μl of HCl (0.3 M). 1 ml of trichloromethane was added and extracted for three times. The aqueous phase was collected and diluted 4 times by deionized water, and passed through a 0.45 μm syringe filter before HPLC analysis. The sample (10 μl) was analyzed by an Shimadzu LC-2030C 3D HPLC system equipped with an UV detector set at 250 nm. The separation was performed on an Agilent Eclipse Plus C18 column (4.6 mm × 250 mm, 5 μm) at a flow rate of 0.8 ml/min at 30°C. The mobile phase was acetonitrile (A) mixed with phosphate-buffered saline (PBS, pH 6.8), 16%–18% (A) for 30 min.

### 2.8 Infrared spectroscopy analysis

In order to identify the fundamental groups present in polysaccharides’ structures, Fourier transforms infrared spectroscopy (FT-IR) analysis is performed. The RFP powder and KBr powder were thoroughly mixed and pressed. The spectrum was determined using an FT-IR spectrophotometer (Thermo Fisher Nicolet Is10, Thermo Fisher Scientific, Waltham, MA, US) between the frequency range of 500–4,000 cm^−1^.

### 2.9 Scanning electron microscopy

SEM is a common technology that provides visual evidence of the shape and surface characteristics of polysaccharides (Lilledahl and Stokke, 2015). An appropriate amount of sample was taken on a conductive silicone tape and evenly poured. After spraying with gold, it was observed with JSM 6701F field emission scanning electron microscope FEI Inspect F50(FSEM) (FEI Co., Hillsboro, Oregon, US).

### 2.10 *In vitro* antioxidant and antibacterial activity

#### 2.10.1 DPPH radical scavenging assay

The method was applied according to the previous report with slight modification (Wang et al., 2017). 200 μl of different concentrations (0.25, 0.5, 1, 2, 4 mg, 8 mg/ml) of the sample solution (in double distilled water) was added to 200 μl DPPH (1 mmol/L in methanol) and vortexed at room temperature for 10 min in the dark. The absorbance was measured by Microplate reader (Biotek, Winooski, VT, United States) at 517 nm. Vitamin C was used as a control drug at the same concentration.

#### 2.10.2 Hydroxyl radical scavenging assay

The procedure was conducted by referring to the previous report with slight modification (Giese et al., 2015). The reaction mixture contained 100 μl FeSO_4_ (9 mM), 100 μl H_2_O_2_ (9.8 mM), 100 μl sodium salicylate (9 mM), and 150 μl of different concentrations (0.25, 0.5, 1, 2, 4 mg, 8 mg/ml) of polysaccharides. After incubation for 0.5 h at 37°C, the absorbance was measured at 510 nm. Vitamin C was used as a control drug at the same concentration.

#### 2.10.3 Superoxide anion radical scavenging activity

The method was applied according to the previous report with slight modification (Li et al., 2021). 3 ml of Tris-HCl buffer (0.05 mol/L, pH = 8.2) and 0.1 ml of different concentrations (0.25, 0.5, 1, 2, 4 mg, 8 mg/ml) of RFP were added to the glass tube. After 20 min of water bath equilibrium at 25°C, 0.3 ml of pyrogallol (7 mM) was added to react accurately for 4 min 1 ml of HCl (10 mol/L) was added to terminate the reaction. Absorption was measured at 420 nm. Vitamin C was used as a control drug at the same concentration.

#### 2.10.4 ABTS + radical scavenging activity

The ABTS^+^ radical scavenging activity assay of RFP was performed by the method according to a previous report with some modifications (Shang et al., 2018). ABTS solution (7 mM) was mixed with an equal volume of potassium persulfate solution (2.45 mM) to form ABTS^+^·cation free radicals, and then placed in a brown reagent bottle, and stored at room temperature for 16 h in the dark. During the experiment, the above ABTS^+^ stock solution was diluted with PBS buffer solution (pH 7.4), placed at 30°C for 30 min, and the absorbance was adjusted to 0.7 ± 0.02 at 734 nm. 100 μl of different concentrations (0.25, 0.5, 1, 2, 4 mg, 8 mg/ml) of the polysaccharide solution (in double distilled water) was mixed with 2 ml ABTS^+^ solution and vortexed at room temperature for 20 min in the dark. Absorption was measured at 420 nm. Vitamin C was used as a control drug at the same concentration.

The scavenging rate above was calculated according to the formula below(d):

Scavenging activity %=[Ao−(Ai−Aj)]/Ao×100 [d]

where Ao is the absorbance of Vitamin C, Ai is the absorbance of polysaccharides, while Aj represents the absorbance of blank control.

#### 2.10.5 NRK-52E cell culture

NRK-52E cells were obtained from Cell Bank of Chinese Academic of Science (Shanghai, China) and grown in high glucose DMEM supplemented with 10% (v/v) heat-inactivated FBS, 100 μg/mL penicillin and 100 μg/mL streptomycin in a 5% CO_2_ humidified incubator at 37°C.

#### 2.10.6 H_2_O_2_-induced model

Cells were incubated in 96-well plates, at a density of 5 × 10⁴ cells/well for 48 h, then the cells were treated with different concentrations of H_2_O_2_ (600, 400, 200, 100, 50, 25, 0 μM) for another 18 h. Then cell viability were measured by MTT assay using MTT kit according to the manufacturer’s protocol. The absorbance of the formazan dye was measured on a microplate reader at a wavelength 570 nm.

#### 2.10.7 RFP protects against H_2_O_2_-induced cytotoxicity of NRK-52E

Cells (5 × 10⁴ cells/well) were seeded in 96-well plates for 24 h, then treated with different concentrations of RFP (5,000, 2,500, 1,250, 625, 312, 0 μg/ml) for another 24 h. H_2_O_2_ was administrated at proper concentration. After H_2_O_2_ exposure for 18 h, cell viability were measured by MTT assay.

#### 2.10.8 Cell apoptosis analyzed by annexin V-FITC/PI double-staining assay

Cells (10 × 10⁴ cells/well) were seeded in 6-well plates for 24 h, then treated with 2,500 μg/ml of RFP for another 24 h. H_2_O_2_ was administrated at proper concentration for 18 h. Cells were collected and washed three times with pre-cold phosphate-buffered saline. Then cells were re-suspended in the binding buffer containing annexin V-FITC and propidiumiodide (PI). After incubation for 20 min at room temperature, BD Facsverse Cell Analyzer (BD Biosciences, San Jose, CA, United States) was used to perform flow cytometry analyses.

### 2.11 RFP on cisplatin-induced acute kidney injury

#### 2.11.1 Animal model

Healthy male Kunming mice (weight 30 ± 2 g) were provided by Chengdu Dashuo Experimental Animal Co., Ltd., Chengdu, China (Experimental Unit Use Permit No. SYXK (Chuan) 2014-124).

Animals were housed at a controlled temperature of 25 ± 1°C and 60 ± 5% humidity under a 12-h light-dark cycle lamp and provided with a diet of freely accessible water throughout the experimental period. All mice were approved by the Experimental Animal Ethics Committee of Chengdu University of Traditional Chinese Medicine with the application filing number 2022-10. After 7 days of acclimation, mice were randomly designed into six groups (8 mice per group): normal control group (Control), model control group (Model), Fer-1 control group (Fer-1), RFP high dose group (RFP-H), RFP medium-dose group (RFP-M), and RFP low dose group (RFP-L). The dosing pattern for each group is shown in Table 1. All groups given by gavage for 10 days (Li et al., 2018). One hour after the end of treatment on day 7, all groups were treated with a single intraperitoneal injection of cisplatin at 20 mg/kg body weight, except for normal control group, which was injected intraperitoneally with 0.9% saline (Zhou et al., 2022).

**TABLE 1:** Types and doses of drugs administered to different groups of mice.

Group	Drug types	Drug administration route	Dosage	Processing time
Control	double distilled water	Oral administration	0.1 ml/10 g	10 days
Model	double distilled water	Oral administration	0.1 ml/10 g	10 days
Fer-1	Fer-1 (in 2% DMSO in saline)	Intraperitoneal injection	1.5 mg/kg	10 days
RFP-H	RFP(in double distilled water)	Oral administration	800 mg/kg	10 days
RFP-M	RFP(in double distilled water)	Oral administration	400 mg/kg	10 days
RFP-L	RFP(in double distilled water)	Oral administration	200 mg/kg	10 days

The mice were executed on day 11, and the serum was collected, centrifuged at 3,500 rpm, 4°C for 10 min, and stored at −20°C for the determination of renal function indexes. The kidneys were also stored at −80°C for further use.

#### 2.11.2 Serum biochemical assay

Blood urea nitrogen (BUN) and blood creatinine (Cr) levels were measured according to the kit manufacturer’s instructions, using mouse serum to complete the assay.

#### 2.11.3 Histological analysis

Isolated kidney tissues were immediately fixed in 4% paraformaldehyde and then dehydrated in graded series of ethanol. After dehydration, the tissues were cleared in xylene and embedded in paraffin. Thin sections were mounted on glass slides and stained with hematoxylin-eosin (H&E) staining.

#### 2.11.4 Tissue biochemical index assay

A variety of biochemical parameters of mouse kidney tissues were also measured according to the instructions of the kit, including: free ferrous ions in tissues (Fe^2+^), MDA, SOD, GSH, GPX-4 and 4-HNE.

### 2.12 Statistical analysis

All data are presented as mean ± SD. The experimental design and analysis of the data were performed using Design-Expert software version 7.0 (Stat-Ease Inc. Minneapolis, United States) and SPSS software 26.0 (SPSS, Chicago, IL). Images were then plotted using GraphPad Prism version 5.01 (GraphPad Software, San Diego, CA) and Flowjo 7.6.2 (Tree Star, Inc. Ashland, OR, United States). Values of *p* < 0.05 were considered statistically significant.

## 3 Results and discussion

### 3.1 RFP extraction process optimization

**FIGURE 1:**
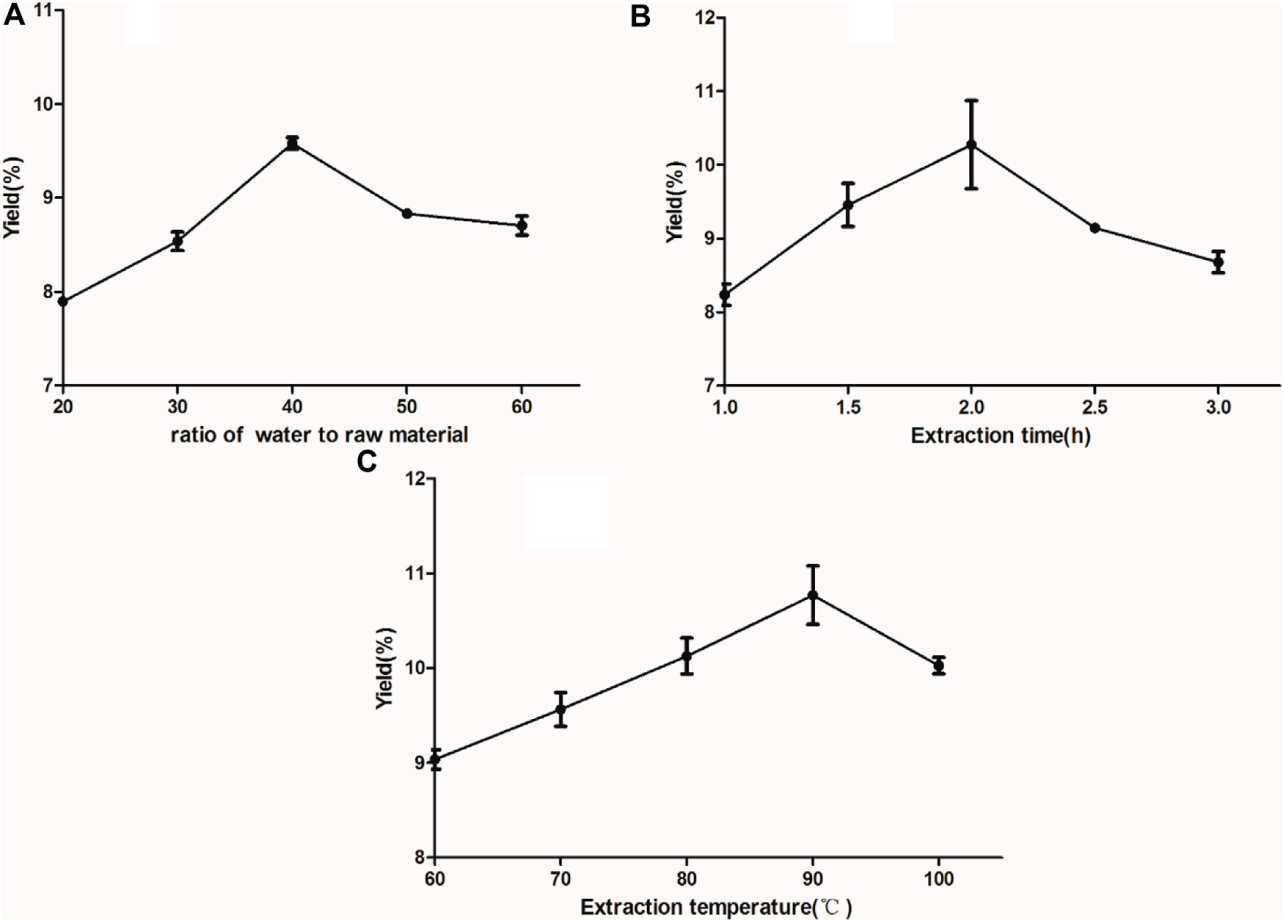
Effect of different feed-to-liquid ratio **(A)**, extraction time **(B)** and extraction temperature **(C)** on the extraction rate of RFP. **(A)** Experiments with different ratios of water to raw material (20:1, 30:1, 40:1, 50:1, 60:1) **(B)** Experiments with different extraction times (1, 1.5, 2, 2.5, 3 h) **(C)** Experiments with different extraction temperatures (60, 70, 80, 90, 100°C).

The results of the single-factor tests are shown in Figure 1 Three independent parameters including A, extraction time (1.5, 2, 2.5 h); B, extraction temperature (80, 85, 90°C); C, ratio of water to raw material (30:1, 40:1, 50:1) were carried out on the basis of single factor tests for BBD to optimise the important variables. Three levels were coded as −1, 0, 1 for low, medium, high levels. The yield of polysaccharides was a response. As shown in Table 2, the yield of polysaccharides ranged from 5.956 to 10.767%. By applying multiple regression analysis on the experimental data, a mathematical model describing the yield (Y) as a function of the coded independent variables was given by the following second-order polynomial equation(a):

Y=10.02+0.29A+0.15B+0.71C−0.32AB+0.091AC−0.76BC−0.83A²−0.99B²−1.23C

**TABLE 2:** Box–Behnken design (coded) and results for extraction yield of RFP.

No.	A (Extraction temperature,°C)	B (Extraction time,h)	C(Ratio of water to raw material)	Yield (%)
1	90	2	30:1	7.741
2	85	2.5	50:1	8.115
3	85	2	40:1	9.462
4	80	2	50:1	7.976
5	85	2.5	30:1	7.898
6	85	2	40:1	10.767
7	90	2	50:1	9.016
8	90	2.5	40:1	8.11
9	80	2	30:1	7.065
10	85	2	40:1	9.582
11	85	1.5	30:1	5.956
12	90	1.5	40:1	8.591
13	85	1.5	50:1	9.198
14	85	2	40:1	10.032
15	80	1.5	40:1	7.642
16	85	2	40:1	10.237
17	80	2.5	40:1	8.427

**FIGURE 2:**
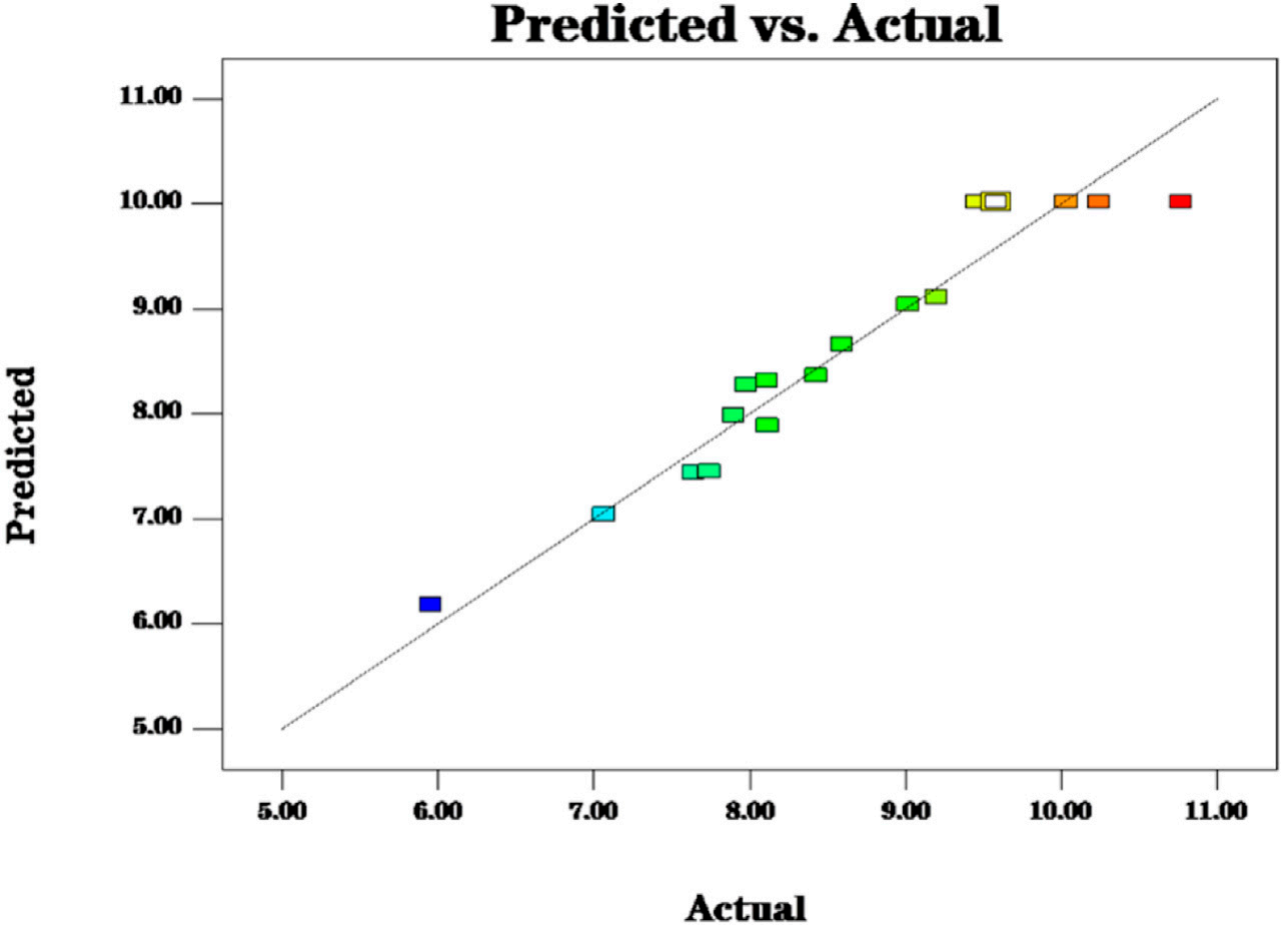
The model predictions are consistent with the actual values and the model predictions are reasonable.

The fit statistics of extraction yield (Y) for the selected quadratic predictive model were shown in Table 3. The high F-value (11.72) and low *p*-value (<0.05) suggested that the regression models are significant. The lack of fit was not significant (F-value = 0.47, *p*-value = 0.7213 > 0.05), indicating that the model is adequate for predicting the yield of polysaccharides (Figure 2). The coefficient (*R* ^2^ = 0.9378) and the adjusted determination coefficient (Adj *R* ^2^ = 0.8578) indicated a high correlation between the predicted and experimental values. High Adequate precision (10.805) indicated adequate signal. These statistical data altogether indicated that the model had shown a good fit with the experimental data. Furthermore, it can be seen from the experimental *p*-value that the three factors in this experiment had different effects on the yield of (Ikeda et al., 2021) polysaccharides, the order is C > A > B, and the order of interaction influence is BC > AB > AC. Among them, C, BC, A^2^, B^2^ and C^2^ had great influence on the extraction rate of polysaccharides (*p* < 0.05).

**TABLE 3:** Analysis of variance (ANOVA) results for response surface optimization of the RFP extraction process.

Source	SS	DF	MS	F-value	*p*-value
Model	22.55	9	2.51	11.72	0.0019
A-extraction temperation	0.69	1	0.69	3.22	0.1156
B-extraction time	0.17	1	0.17	0.79	0.4033
C-ratio of water to raw material	3.98	1	3.98	18.63	0.0035
AB	0.4	1	0.4	1.87	0.2133
AC	0.033	1	0.033	0.15	0.7056
BC	2.29	1	2.29	10.7	0.0136
A^2^	2.92	1	2.92	13.66	0.0077
B^2^	4.13	1	4.13	19.33	0.0032
C^2^	6.41	1	6.41	29.98	0.0009
Residual	1.5	7	0.21		
Lack of Fit	0.39	3	0.13	0.47	0.7213
Pure Error	1.11	4	0.28		
Cor Total	24.05	16			

Notes: SS-Sums of squares; DF-Degree of freedom; MS-Mean squares.

A three-dimensional (3-D) response surface plot and a contour plot are drawn that provide a visual interpretation of the interaction between the two test variables and the relationship between the response of each variable and the experimental level.

**FIGURE 3:**
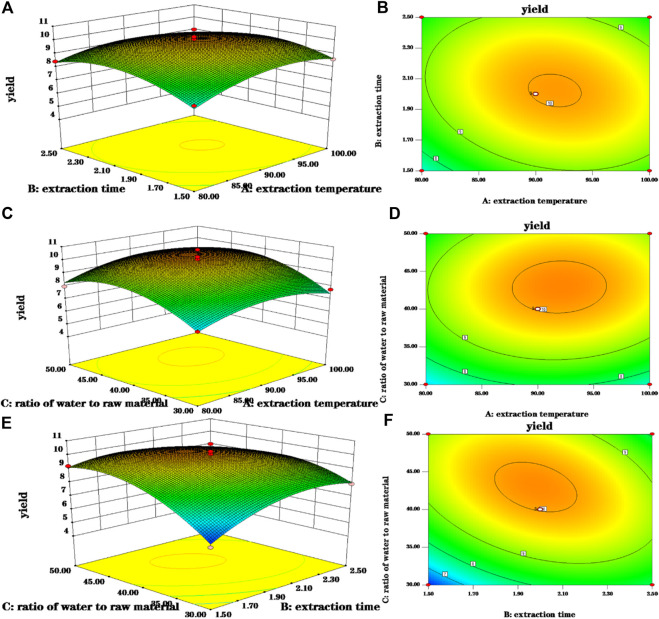
3-D response surface and 2-D contour plots for polsaccharides yield. **(A,B)** Were 3-D response surface and 2-D contour plots showing the interaction of extraction time and extraction temperature on the yield of polysaccharides; **(C,D)** were 3-D response surface and 2-D contour plots showing the interaction of ratio of water to raw material and extraction time on the yield of polysaccharides; **(E,F)** were 3-D response surface and 2-D contour plots showing the interaction of ratio of water to raw material and extraction time on the yield of polysaccharides.

Elliptical contours were obtained when there was a significant interaction between the independent variables while circular contour plots indicated otherwise. According to Figures 3B,D,F, the shape of its contours were different, indicating the interaction between C and B was more significant than the interaction between C and A, and interaction between A and B. This result was similar to their *p*-values in Table 2. The optimum extraction conditions for maximum extraction yield of polysaccharides proposed by the Design-Expert software were as follows: A of 92.09°C, B of 1.96 h, C of 43.19. Considering the convenience during actual operation, the actual extraction conditions were modified slightly: A of 90°C, B of 2 h, C of 43. To verify the adequacy of the optimal condition, three verification tests were carried out under these conditions, and the actual yield of polysaccharides obtained was 10.130 ± 0.041%, which highly matched the yield (10.153%) predicted by the regression model. The results indicated that the model was accurate and adequate in predicting polysaccharides extraction conditions. Ma et al. (2005) showed the best extraction process was to add 150 ml water, repeat 3 times, 90 min of each time, and the glucose content of polysaccharides was 56.71%. The temperature and solid-liquid ratio were the main factors affecting the yield of crude polysaccharides from Fuzi. The optimum extraction process was solid-liquid ratio of 1:40, extraction temperature of 90°C, extraction duration of 2 h, pH 8, alcohol concentration of 80% (Shu et al., 2006). Ye et al. (2013) found the best extraction process was to add 10 times the amount of water, soak for 30 min, decoct 2 times, each time 2 h. And the obtained polysaccharides had sugar content of 15%. Other researchers had used microwave extraction to determine the extraction process of polysaccharides and the results showed the optimal process was the ratio of material to liquid of 1:20 at 80°C for 10 min. Under this condition, the polysaccharides extraction rate was 16.10% (Zhu et al., 2015). Ultrasonic extraction was applied as well. The ultrasonic extraction process was finally optimized by the uniform design method. The liquid-to-liquid ratio was 10, the ultrasonic time was 34 min, the ultrasonic temperature was 73°C, and the yield of polysaccharide was as high as 19.05% (Lu and Niu, 2017). Because the biological enzymatic extraction has the advantages of mild and non-toxic reaction conditions, some scholars have applied it to the extraction of Fuzi polysaccharides (Alexandre et al., 2019). The optimal extraction conditions were extraction temperature of 45°C, enzyme dosage of 1.4%, extraction time of 1.5 h, pH 4.5, and the actual yield of polysaccharide was 15.77% (Zhang, 2016). From the results of the previous and our experiments, it can be seen that the main factors affecting the extraction of polysaccharides from Fuzi are temperature and ratio of water to raw material. In order to facilitate the subsequent detection, the aconite polysaccharide was freeze-dried into powder, and some amounts of polysaccharides may be lost during the process. In addition to the heating and reflux method, microwave and ultrasonic extraction methods are also applied which seems has high yield. However, given that Fuzi contains toxic components, the heating method is selected. Taken together, in the next step, the same batch of Fuzi should be taken, and the effect of different extraction methods on yield of polysaccharide should be investigated in one experiment.

### 3.2 Chemical analysis and molecular weight of RFP

**FIGURE 4:**
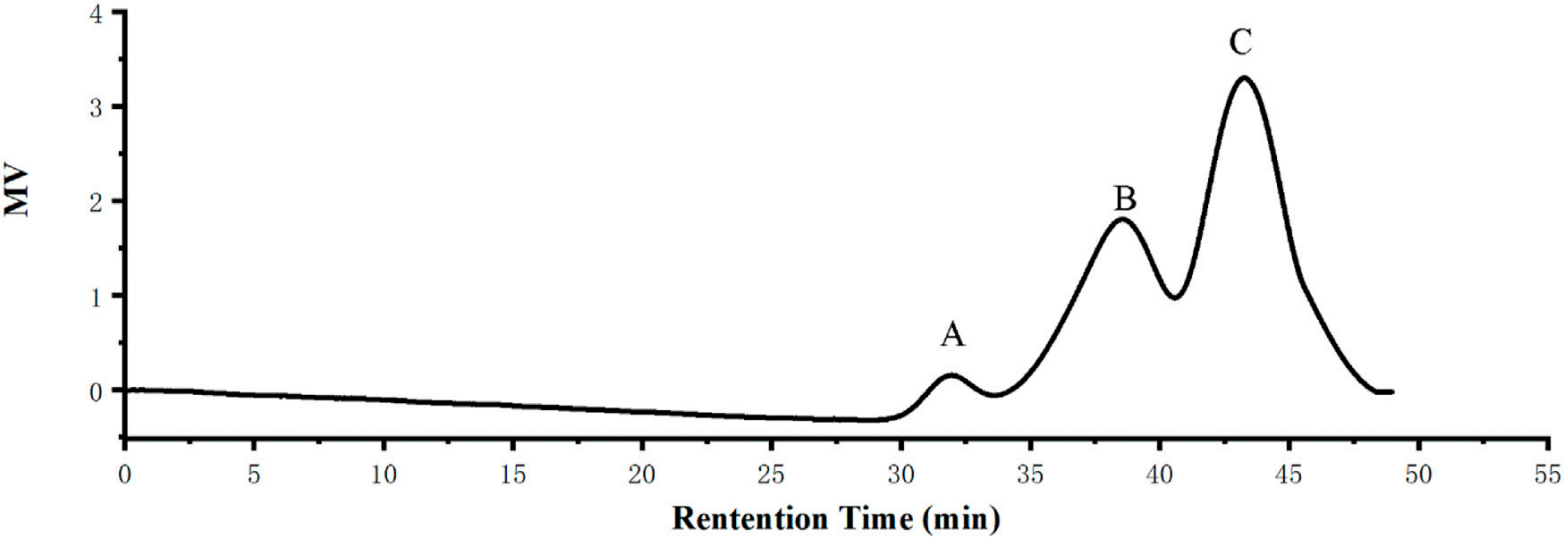
RFP consists of three polysaccharides of different molecular weights. **(A)** Molecular weight of 1,211.136 kDa with a peak area of 4.6% (w/w) **(B)** Molecular weight 64.677 kDa, 37% peak area (w/w) **(C)** Molecular weight 7.973 kDa, peak area 58.4% (w/w).

The results showed that the total carbohydrate content of the RFP was 85.85% + 3%; the protein amount was 2.05% + 2.01%; and the glyoxylate rate was 5.68 + 6.23%. Molecular weight is considered as a significant structural feature of polysaccharides in their structure-function relationships. The results of the molecular weight determination of RFP were summarized in Figure 4 consisting of three components with different molecular weights, including a polysaccharide containing 58.4% (w/w) of the peak area with a molecular weight of 7.973 kDa, 37% (w/w) with a molecular weight of 64.677 kDa, and 4.6% with 1,211.136 kDa. The large variation in average molecular weight of RFP facilitates subsequent isolation studies (Spadoni Andreani et al., 2021).

### 3.3 Monosaccharide composition of FRP

**FIGURE 5:**
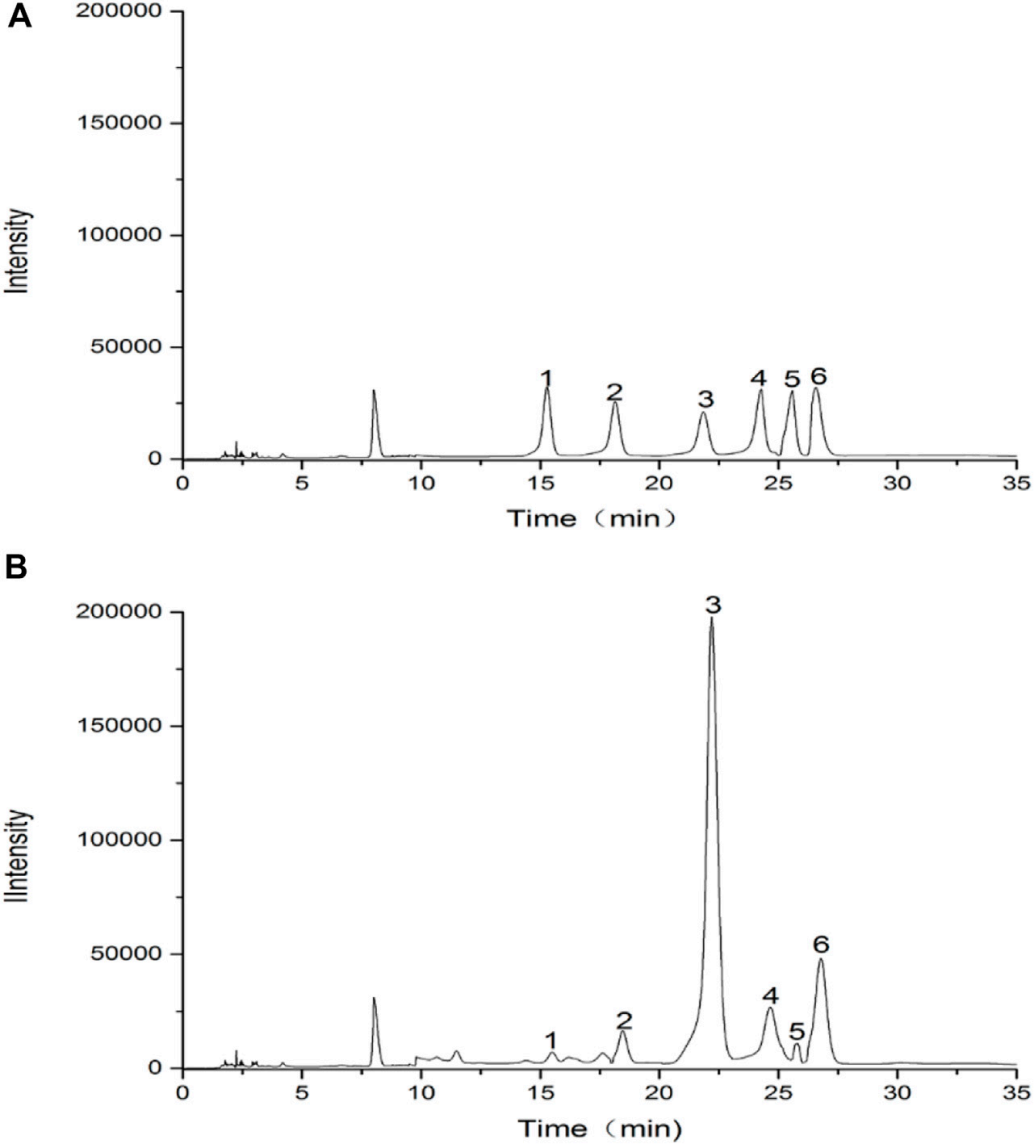
HPLC chromatograms of hydrolysed polysaccharide derivatives and mixed monosaccharide standard derivatives. **(A)** HPLC chromatogram of monosaccharide standards; **(B)** HPLC chromatogram of RFP. (1) rhamnose, (2) D-galacturonic acid, (3) D-glucose, (4) D-galactose, (5) xylose, (6) L-arabinose.

Results of the monosaccharide composition of FRP were shown in Figure 5. It was found that RFP consisted of rhamnose, D-galacturonic acid, D-glucose, D-galactose, xylose, L-arabinose with a molar ratio of 1: 2.34: 59.12: 4.64: 1.88: 10.72. The monosaccharide compositional analysis implied that glucose is the main monosaccharide in FRP. Previous research showed polysaccharide from Fuzi was composed of mannose, glucose, rhamnose, galactose, xylose, fucose and arabinose, which mainly were glucose by high performance liquid chromatography coupled with electrospray ionization multi-tandem mass spectrometry (HPLC/ESI-MSn) (Wang et al., 2016). Four glycans from Aconitum carmichaeli Roots were isolated and aconitaned B, C, and D all contained rhamnose, arabinose, mannose, galactose and glucose with different molar ratio. However, glucuronic acid was only present in aconitan C. These different structures led to different hypoglycemic activity (Konno et al., 1985). Therefore, it is crucial to identify the monosaccharide residue type, sugar chain sequence, sugar residue linkage mode, degree of polymerization, configuration of glycosidic bond by GC-MS, HPLC-MS, NMR. However, LC-MS based methods have some limitations including long separation times, low resolution of oligosaccharide mixtures, incompatibility of eluents, and often require oligosaccharide derivatization. Recent years, capillary electrophoresis-mass spectrometry were applied because it could offer rapid analysis of complex glycosaminoglycan mixtures, providing detailed structural and quantitative data (Sun et al., 2016).

### 3.4 FT-IR spectroscopy analysis

**FIGURE 6:**
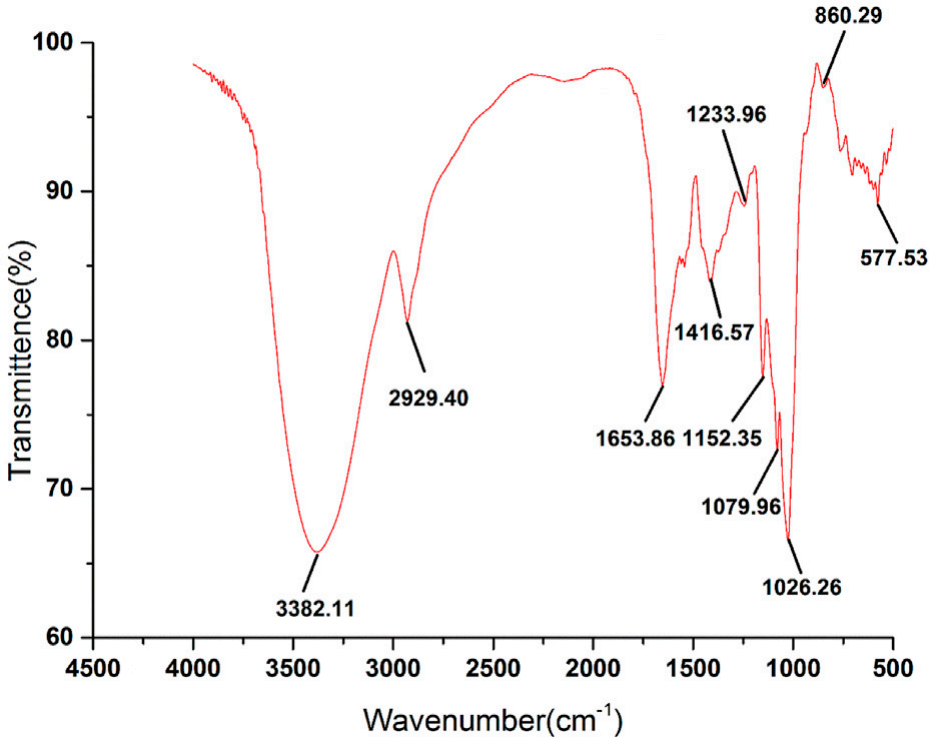
FT-IR spectra of RFP. RFP has a significant polysaccharide profile and β-pyranose conformation.

As a commonly used analytical method in the identification of primary structure of polysaccharides, FT-IR can be used to analyze the glycosidic bond type, type of functional group and sugar ring configuration of polysaccharides (Chen et al., 2016). The FT-IR spectrum of RFP was shown in Figure 6. The strong absorption peak near 3,382.11 cm^−1^, the weak absorption peaks at 2,929.40 and 1,653.86 cm^−1^ were caused by the stretching vibration of the hydroxyl groups, C-H and combined water on the sugar chain, respectively (Lu et al., 2022; Yang et al., 2022). Those characteristic absorption peaks were often used to determine whether the sample was polysaccharides. In addition, the absorption peak at 1,233.96 cm^−1^ was the angular vibration of C-H. The peak at 1,653 cm^−1^ represents a C-O bending vibration, and the peak at 1,416 cm^−1^ is caused by a C-H bending vibration, representing the presence of a glyoxylate component (Ma et al., 2022; Yi et al., 2022), and the band near 1,079 cm^−1^ is caused by symmetric stretching vibrations of the furan ring. There was an absorption peak at 860.29 cm^−1^, indicating that the polysaccharide contained the β-pyranose configuration of glucose. These characteristic IR absorption peaks indicate that RFP has a typical glycosyl group, contains glyoxylates and also includes the beta conformation of pyranose. Previous studies also confirmed that. (Yang et al., 2020).

### 3.5 SEM analysis of RFP

**FIGURE 7:**
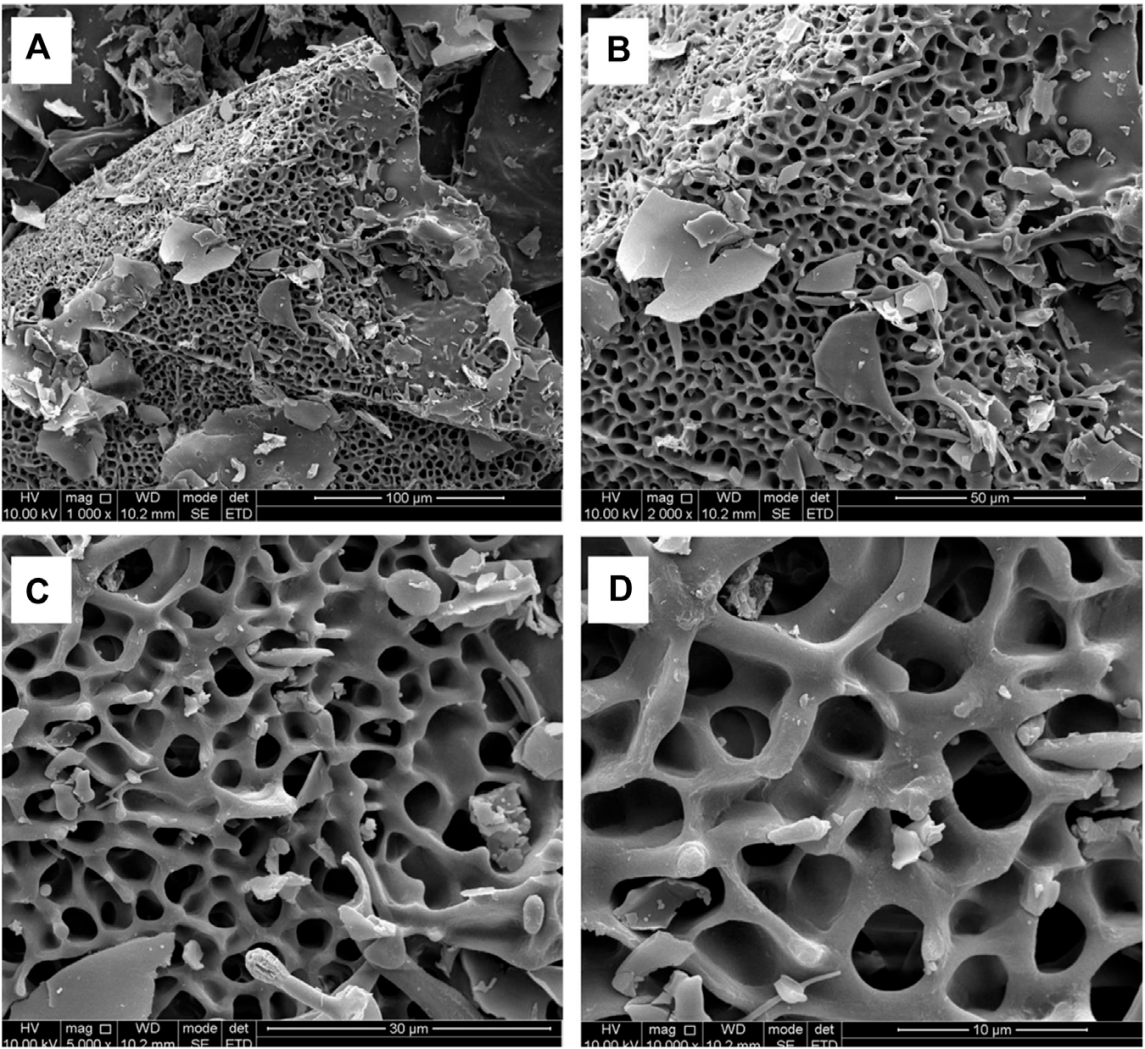
SEM observations show that RFP has a honeycomb network structure with tight and regular cross-linking. **(A)** Magnification at 1,000 (scale bar is 100 μm) **(B)** Magnification at 2000 (scale bar is 50 μm) **(C)** Magnification at 5,000 (scale bar is 30 μm) **(D)** Magnification at 10,000 (scale bar is 10 μm).

The microstructure of the RFP was investigated by SEM at different magnifications (1,000×, 2000×, 5,000× and 100,00×) (Figure 7). Under the electron microscope of 1,000 magnification, irregular slices, fragments, large flakes, a small number of short rods and spheres were observed, and the structure was loose and unevenly distributed. Among them, an irregular honeycomb body having a length of about 200 μm and a width of about 300 μm was observed. Further, it was observed under the electron microscope of 2,000 and 5,000 magnification that the honeycomb network structure was closely and regularly crosslinked. At 10,000 magnification, the surface was smooth and the structure was closely crosslinked, which may be related to the strong interaction between molecules (Lu et al., 2019).

### 3.6 *In vitro* antioxidant activity of RFP

**FIGURE 8:**
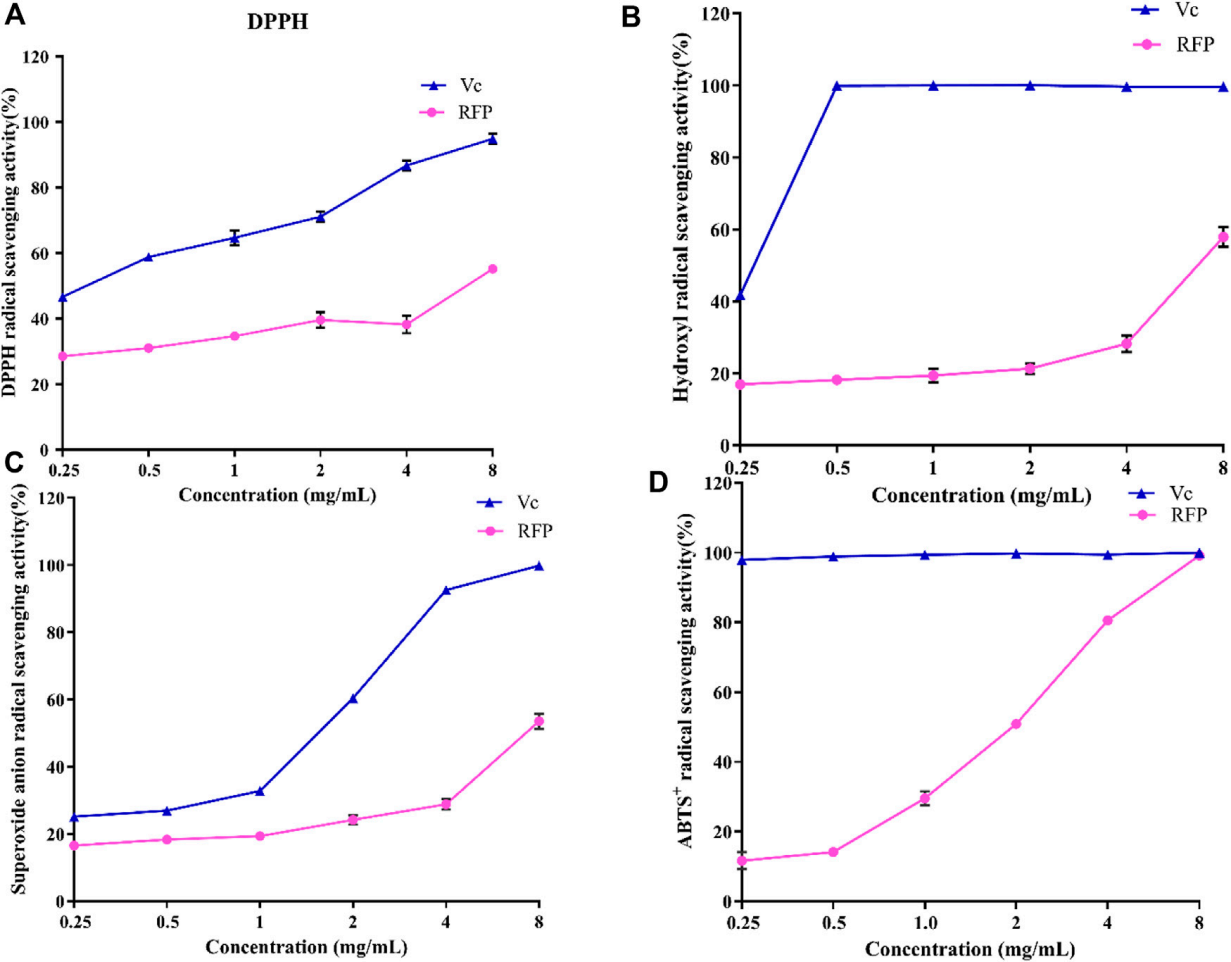
Antioxidant activities of RFP and Vc. **(A)**DPPH radical scavenging assay; **(B)** hydroxyl radical scavenging assay **(C)**superoxide anion radical scavenging assay; **(D)** ABTS^+^ radical scavenging assay. Each value is represented as the mean ± SD (n = 3)

Then, bioactivities of RFP were studied. Firstly, we examed its antioxidant property. As shown in Figure 8, the ability of RFP to scavenge DPPH, hydroxyl, superoxide anion and ABTS + radicals was compared with Vitamin C, a known antioxidant compound. RFP could scavenge these radicals in a dose-dependent manner with concentration ranged from 0.25 to 8 mg/ml. With dose of 8 mg/ml, Vitamin C exhibited approximately 100% scavenging activity. In Figure 8A, the maximum DPPH radical scavenging activity of RFP was with 8 g/ml, and corresponded to 55.16 ± 1.02%. Thus, the results indicated that RFP possessed scavenging activity in DPPH-radicals, though RFP had weaker capacity when compared with Vitamin C (*p* < 0.05). As shown in Figure 8B, it showed a relatively low effect on hydroxyl radicals of 16.92 ± 0.84% at 0.25 mg/ml. The scavenging rate of hydroxyl radical increased slowly with the increase of concentration, and reached the maximum value of 57.90 ± 3.85% at 8 mg/ml. However, the inhibitory effect of Vitamin C drastically increased to 100% at 0.5 mg/ml. Therefore, RFP exhibited a moderate scavenging effect on hydroxyl radicals. Based on the results shown in Figure 8C, the maximum scavenging superoxide anion radical activity of RFP is 53.56 ± 3.24% when treated with 8 mg/ml while Vitamin C showed remarkable scavenging activity against superoxide anion radicals (*p* < 0.05). Thus a conclusion could be drawn that RFP possessed moderate scavenging effects on superoxide anion radicals. The effects of RFP on ABTS + radical scavenging abilities were shown in Figure 8D. Its 50% inhibiting concentration was 1.724 mg/ml. It totally inhibited the activity with the concentration of 8 mg/ml at which showed no significant difference between RFP and Vitamin C (*p* > 0.05). Therefore, it can be concluded that RFP had a strong scavenging effect on ABTS + radicals. Within the selected concentration range, the higher concentration, the greater effect of RFP on scavenging those radicals. However, the scavenging activities varied for different radicals. As for DPPH, hydroxyl, superoxide anion radicals, RFP only showed moderate scavenging effects around 50%, lower than Vitamin C (*p* < 0.05). Due to the reduction of phenolic hydroxyl groups in polysaccharides during extraction or purification, which affects the pairing of hydrogen free radicals with DPPH free electrons, the scavenging effect of RFP on DPPH radicals were reduced. Perhaps it is due to the fact that some of the components which combined with polysaccharides in the process of separation and purification have not been completely removed, resulting in poor scavenging capacity of hydroxyl, superoxide anion radicals. This supposition is consistent with the results of previous studies of polysaccharides from Lentinus edodes (Yin et al., 2018). However, RFP showed great scavenging effects on ABTS^+^ radicals which is equal to Vitamin C with concentration of 8 mg/ml. Similar to this result, Ye et al. (Zhang, 2016) also found that the same concentration of polysaccharides had a higher clearance rate for ABTS radicals than that of DPPH, probably because DPPH radicals would dissolve some O_2_ in the reaction solution and then reacted weakly with O_2_^−^. Consequently, the degree of color change of the reaction liquid was lowered, so that the antioxidant activity appeared to be relatively low. In addition, DPPH radicals are easily interfered with by substances such as pigments, which reduce their sensitivity. Past studies have shown that plant polysaccharides are effective antioxidants that scavenge free radicals. Various factors such as the polarity, monosaccharide composition and water solubility of natural polysaccharides have been found to influence the antioxidant activity of polysaccharides (Yarley et al., 2021). It was found that the antioxidant activity of polysaccharide may be related to its monosaccharide composition. Thambiraj et al. (2015) found that the more galactose content in polysaccharide samples, the stronger free radical scavenging activity. Xu et al. (2011) isolated four different polysaccharides from Angelica dahurica exhibiting different antioxidant activities, with the polysaccharides with higher free radical inhibition capacity having significantly more rhamnose content. In addition, the glyoxylate content (Xu et al., 2019) also increased the antioxidant activity of the polysaccharides, mainly by affecting the glycosidic bonding of the polysaccharides. A study by Yao et al. (2016) suggested that xylose (Xyl), a monosaccharide component, may also play an important role in antioxidant activity.

### 3.7 Impact of RFP

#### 3.7.1 Effect on the apoptosis of NRK-52E cells

**FIGURE 9:**
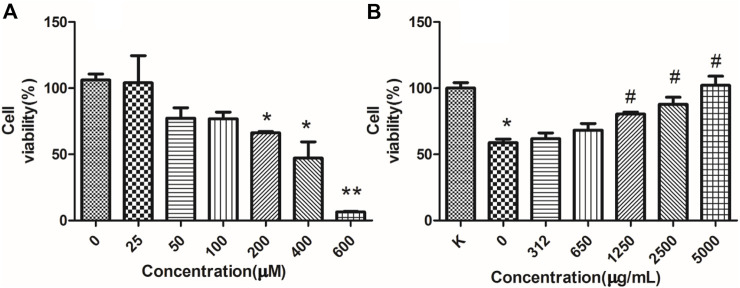
RFP protect against H_2_O_2_-induced cytotoxicity. **(A)** H_2_O_2_-induced cytotoxicity. NRK-52E cells were treated with different concentrations of H_2_O_2_ as indicated for 18 h. **(B)** RFP inhibited the cell death induced by H_2_O_2._ NRK-52E cells were pretreated with different concentrations of RFP for 24h, then exposed with H_2_O_2_ for 18 h. The cell viability was assessed by MTT (‾x± SD, n = 3). *p < 0.05, **p < 0.01 as compared to the control. ^#^ *p <* 0.05 as compared to cells only treated with H_2_O_2_, without RFP solutions.

**FIGURE 10:**
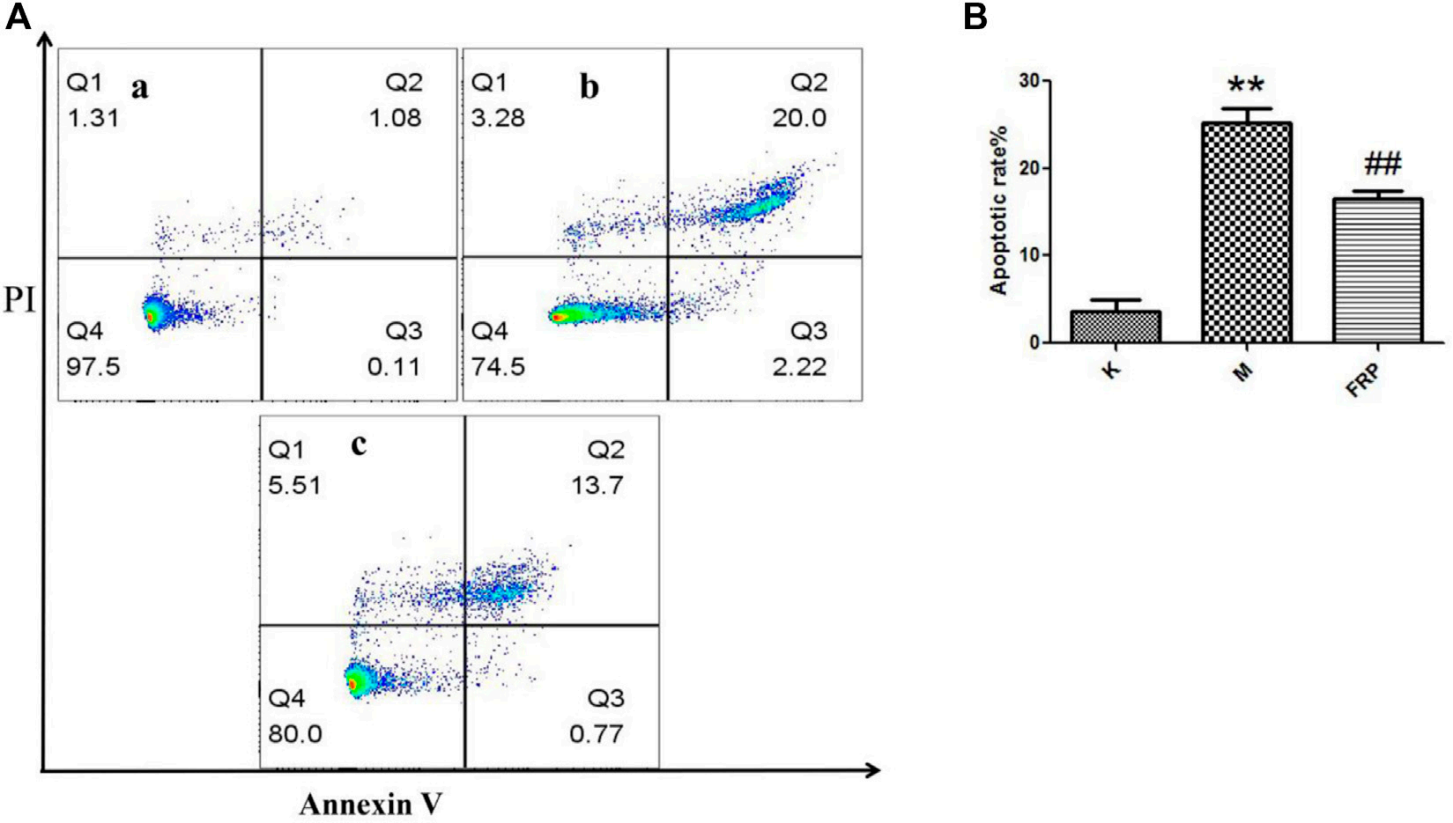
RFP inhibited the cell apoptosis induced by H_2_O_2_. **(A)** Normal cells, **(B)** Cells were exposed to 200 μM of H_2_O_2_ for 18h, c) Cells were pretreated with 2,500 μg/ml of RFP for 24 h, then induced by 200 μM of H_2_O_2_ for 18 h. Apoptotic cells were quantified by flow cytometry after stained with FITC-conjugated Annexin V and PI (‾x±SD, n = 3). The images were a representative of three independent experiments. **p < 0.01 as compared to the control,^##^ *p* < 0.01 as compared to the model group.

Furthermore, its anti-apoptotic effect was studied in NRK-52E cells. To determine the proper concentration of H_2_O_2_, cell viability was measured. As shown in Figure 9A, 25–600 μM of H_2_O_2_ inhibited the proliferation of NRK-52E cells in a concentration-dependent manner after 18 h’exposure. The IC_50_ and IC_35_ were 261.6, 201.3 μM respectively. Then 200 μM were chosen for further study. As shown in Figure 9B, 5,000, 2,500, 1,250 μg/ml of RFP could inhibit the cytotoxic effect of H_2_O_2_, and promoted viability to 80%–100% when compared with model group (only with H_2_O_2_ exposure), while 625 and 312 μg/ml showed no obvious effect. It can be seen from Figure 10 that H_2_O_2_ obviously induced apoptosis in NRK-52E with apoptotic rate of 25.26 ± 2.86% when compared with normal cells (3.68 ± 2.20%). After treated with RFP, the cell apoptotic rate was reduced to 16.53 ± 1.64%. These results suggested RFP could protect NRK-52E cells from the apoptosis caused by H_2_O_2_. Investigation of Yang and Wang turned out Fuzi had the effect of reducing urinary protein, lowering urine nitrogen, creatinine in serum, and reducing kidney damage in adriamycin induced nephropathy in rats (Li et al., 2012). Aristolochic acid was used to establish the chronic renal failure. In this model, the redox state of the kidney was low and disturbed, and uric acid metabolism became abnormal. Fuzi can exert therapeutic effect on aristolochic acid induced chronic renal failure (Du et al., 2012). Fan et al. (2011) found Fuzi could achieve the balance between the content of lactic acid and activity of lactate dehydrogenase in order to protect kidney. Polysaccharides derived from *Pao-Tian-Xiong*, one of the product of Fuzi showed obvious protective effect against chronic renal failure in mice. According to results, polysaccharides alleviated pathological changes in kidneys involving glomerular necrosis, infiltration of inflammatory cells, necrosis of renal tubules (Wang et al., 2019). NRK-52E, kidney tubule epithelial cells, are often used for nephrology. It has been reported that H_2_O_2_ can induce proximal tubule damage that is associated with the induction of various modes of cell death including necrosis, apoptosis and apoptotic oncosis, and the activation of endonuclease. Wang and their results showed H_2_O_2_ induced apoptosis in NRK-52E in a dose dependent manner (100, 300, 500 μM) within 24 h, which was associated with activation and up-regulation of the JNK activity (Wang et al., 2002). Exposure with 250 μM of H_2_O_2_ for 24 h can induce apoptosis in NRK-52E, and this effect can be reversed by over-expression of Peroxiredoxin 1 *via* inhibiting the p38 MAPK pathway (Mei et al., 2015). Oxidant-induced apoptosis generated by H_2_O_2_(300 μM, E24 h) also involved ER stress signaling and CHOP expression. The 24 h exposure to H_2_O_2_ markedly increased the cytotoxicity along with the increased concentrations (200, 400, 600, 800, 1,000 μM) through lipid peroxidation (Katsoulieris et al., 2010). The present study also found H_2_O_2_ obviously induce toxicity in NRK-52E cells. However, 200 μM for 18 h was chosen for further study which is different from above reports, maybe due to different state of cells, incubating environment or reagent. Under the circumstance, RFP clearly attenuated H_2_O_2_-induced toxicity, which could provide RFP as potential natural agent to treat renal diseases.

### 3.8 Protective effect against cisplatin-induced renal injury

#### 3.8.1 Serum biochemical assay

**FIGURE 11:**
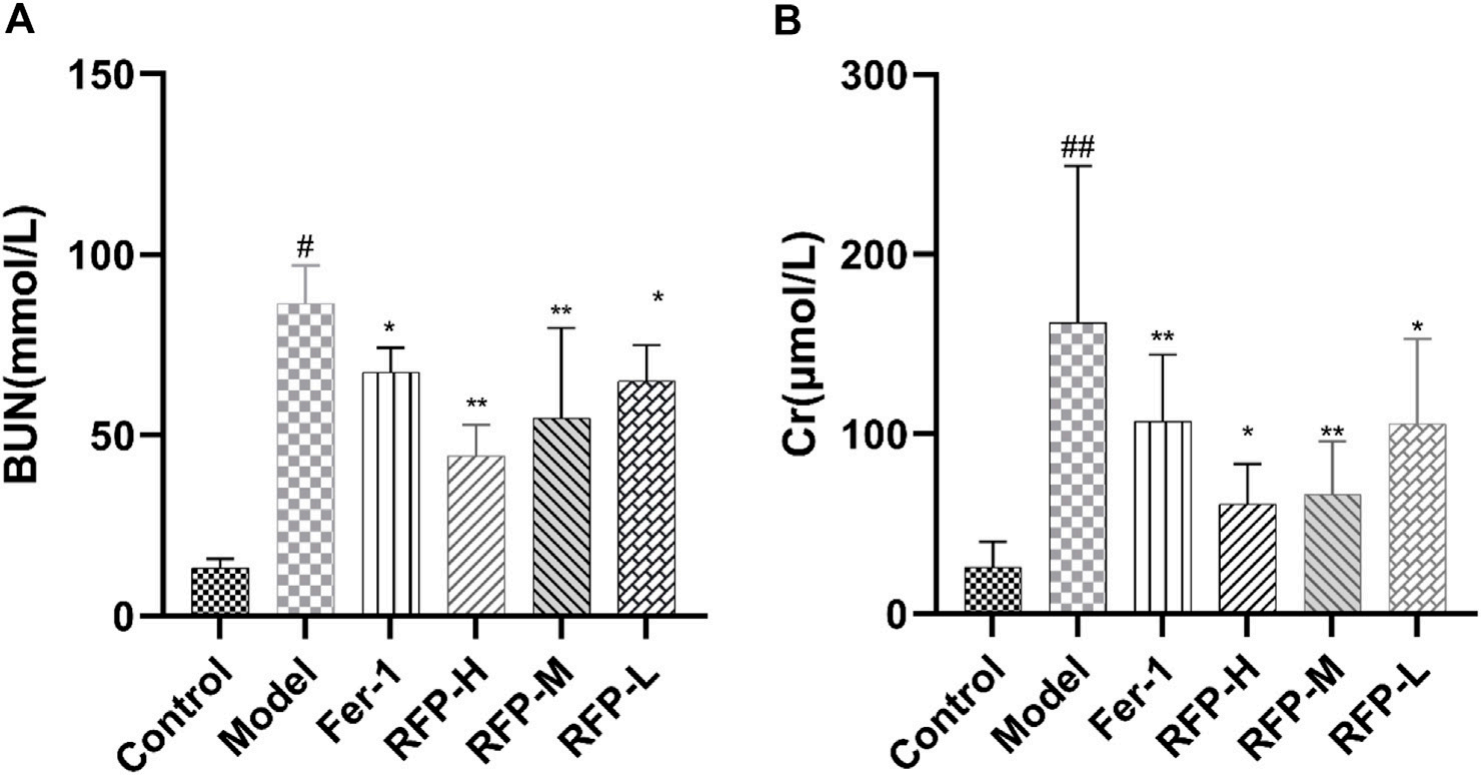
Effect of RFP on the serum levels of BUN and Cr in mice with cisplatin-induced acute kidney injury. **(A)** Serum levels of BUN in different groups of mice; **(B)** Serum levels of Cr in different groups of mice. Data are expressed as mean ± SD (n = 8), ^*^ *p* < 0.05, ^**^ *p* < 0.01as compared to the model group, ^##^ *p* < 0.01 as compared to the blank group.

In accordance with a previous study, serum BUN and Cr levels were measured to determine renal function, and the results are shown in Figure 11(Zhao et al., 2021). The results showed that cisplatin caused severe renal impairment, while Fer-1 and RFP-H significantly reduced the impairment of the kidney function.

#### 3.8.2 Histological analysis

**FIGURE 12:**
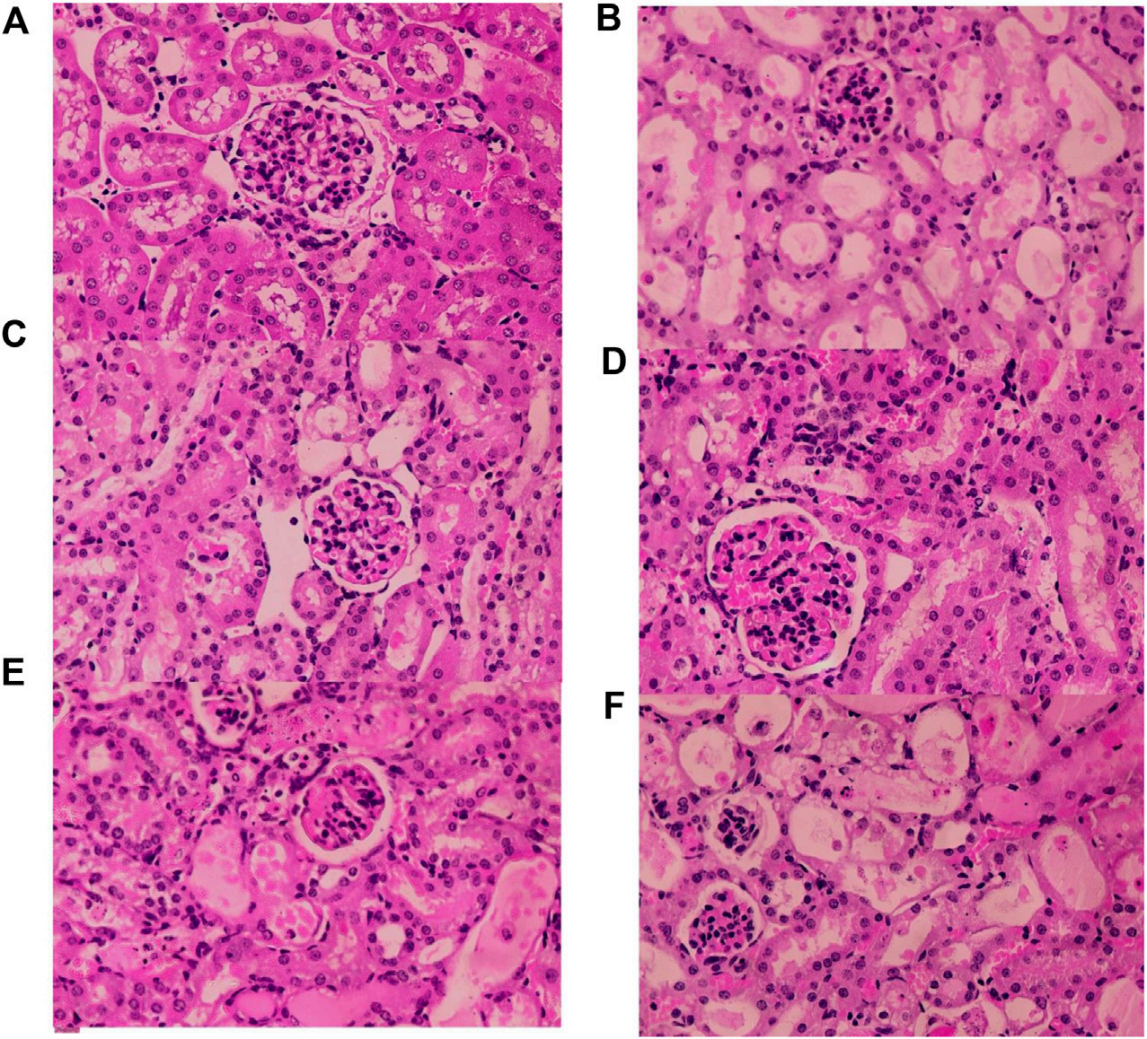
Results of histopathological HE staining of the kidneys of various groups of mice (HE × 400). **(A)** Normal control group **(B)** Model control group **(C)** Fer-1 control group **(D)** RFP high dose group **(E)** RFP medium-dose group **(F)** RFP low dose group.

As shown in Figure 12, no pathological changes were observed in the kidney tissue of the mice in the Control group. In the Model group, renal tissue was more severely damaged, with tubular dilatation or atrophy, vacuolation, detachment and necrosis of tubular epithelial cells. Compared with the Model group, the Fer-1 group showed significantly less tubular necrosis and significantly less tubular dilatation. Compared with the Model group, the RFP-H group showed less tubular epithelial cell necrosis and better tubular structure, while the RFP-M and RFP-L groups showed some reduction in the above pathological changes. Overall, RFP administration reduced renal pathological damage in mice with cisplatin-induced acute kidney injury.

#### 3.8.3 Tissue biochemical index assay

**FIGURE 13:**
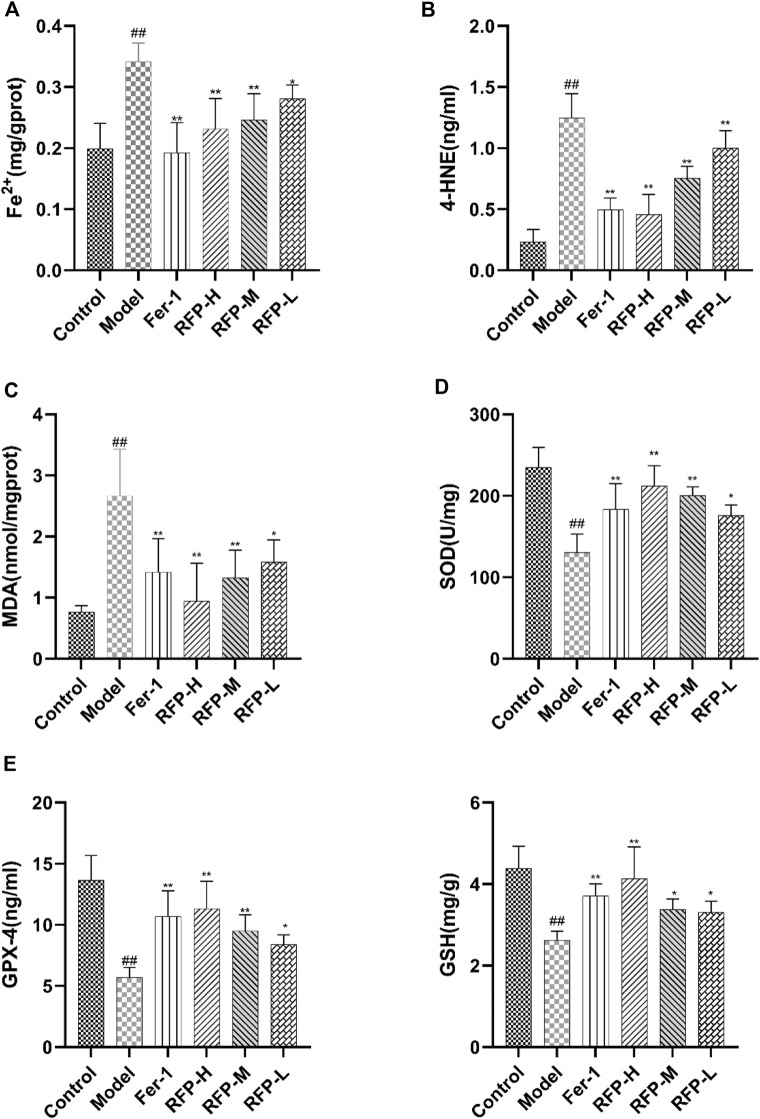
Effect of RFP on the content of six biochemical indicators in renal tissue homogenates of mice with cisplatin-induced acute kidney injury. **(A)** Levels of free ferrous ions content in renal tissue. **(B)** Levels of 4-HNE in renal tissue. **(C)** Levels of MDA in renal tissue. **(D)** Levels of SOD in renal tissue. **(E)** Levels of GPX-4 in renal tissue. **(F)** Levels of GSH in renal tissue. Data are expressed as mean ± SD (n = 8), ^*^ *p* < 0.05, ^**^ *p* < 0.01as compared to the model group, ^##^ *p* < 0.01 as compared to the blank group.

First, we detected an increase in free ferrous ions content after cisplatin treatment in mouse kidney tissue, which was alleviated by Fer-1 and RFP treatment (Figure 13A). Lipid peroxidation is an important marker of ferroptosis (Ikeda et al., 2021; Kim et al., 2021). By measuring 4-HNE (Figure 13B), MDA (Figure 13C), and SOD (Figure 13D), it was found that cisplatin-induced an increase in 4-HNE, MDA levels and a decrease in SOD levels in renal tissues, while treatment with Fer-1 and RFP significantly decreased 4-HNE, MDA levels and significantly increased SOD amounts. This indicated that FRE could improve lipid peroxidation in cisplatin-induced acute kidney injury in mice. In addition, GSH (Figure 13F) and GPX-4 (Figure 13E) levels were measured, and glutathione deficiency or inhibition of the glutathione-dependent antioxidant enzyme GPX-4 was also a key feature of ferroptosis. The data revealed that cisplatin decreases GSH and GPX-4 levels in tissues, while treatment with Fer-1 and RFP significantly elevated GSH and GPX-4 levels. The present study was consistent with previous studies showing that RFP alleviates cisplatin-induced acute kidney injury.

Acute kidney injury (AKI), a common and serious clinical kidney syndrome with high incidence and mortality. Many studies have shown that lipid peroxidation occurs after acute kidney injury (Feng et al., 2022). The concept of ferroptosis was first proposed in 2012 by Dixon et al. (2012). In past studies on iron toxicity, features of iron toxicity were found to include accumulation of lipid peroxides, disturbances in iron metabolism, accumulation of ROS and the consumption of GPX4 and system Xc- (a cysteine/glutamate antiporter system). (Ni et al., 2022). Biochemically, ferroptosis mainly includes the consumption of glutathione (GSH) and the decreased activity of GPX4 (Zheng and Conrad, 2020; Obeng, 2021). The results of the present study showed that in cisplatin-induced kidney injury in mice, renal tissue showed increased Fe^2+^ content, decreased SOD, GSH, and GPX-4 content, and increased MDA, 4-HNE content. This demonstrated that cisplatin-induced acute kidney injury induced the development of ferroptosis. Previous studies have shown that resveratrol protects against cisplatin-induced acute kidney injury by improving MDA, GSH, and endogenous antioxidant enzyme activity and improving the *in vivo* metabolism of cisplatin (Darwish et al., 2018; Ibrahim et al., 2018). Guo et al. found that ginsenoside Rg1 inhibited ferroptosis by reducing iron accumulation and inhibiting lipid peroxidation (Guo et al., 2022). In the present study, we demonstrated that RFP could effectively reduce lipid peroxidation production while increasing the level of the antioxidant enzyme GSH in a cisplatin-induced mouse model of acute kidney injury, suggesting that RFP could alleviate cisplatin-induced oxidative damage induced by acute kidney injury. Notably, we observed that RFP effectively reduced intracellular iron content, increased GPX4 expression and decreased 4-HNE expression. Based on these studies, we speculate that the antioxidant activity of irisin against SA-AKI may be related to the inhibition of ferroptosis. Different doses of RFP exerted different effects, although the results were not statistically significantly different. In conclusion, RFP attenuated cisplatin-induced acute kidney injury in mice, and the mechanism may be related to the inhibition of ferroptosis.

## 4 Conclusion

The extraction, characterisation and activity of RFP were investigated for its exploitation. RFP consists of three polysaccharides with different relative molecular masses of 7.973, 64.677, and 1,211.136 kDa, respectively. It was found that RFP consisted of rhamnose, D-galacturonic acid, D-glucose, D-galactose, xylose, L-arabinose with a molar ratio of 1: 2.34: 59.12: 4.64: 1.88: 10.72. It has a honeycomb structure with significant β-pyranose structure and typical acidic carbohydrate functional group uptake. As a plant polysaccharide, RFP also exhibits significant antioxidant activity and protects against hydrogen peroxide-damaged NRK-52E cells and cisplatin-induced acute kidney injury in mice. In conclusion, this study provides a good strategy for the extraction of polysaccharides from Fuzi and a scientific basis for the development of using it as antioxidants agents. However, more investigations are needed to purify the polysaccharide and to reveal the relationship between structure and biological activity.

## Data Availability

The original contributions presented in the study are included in the article/supplementary material, further inquiries can be directed to the corresponding authors.
